# The ethical issues regarding consent to clinical trials with pre-term or sick neonates: a systematic review (framework synthesis) of the empirical research

**DOI:** 10.1186/s13063-015-0957-x

**Published:** 2015-11-04

**Authors:** E. Wilman, C. Megone, S. Oliver, L. Duley, G. Gyte, J. M. Wright

**Affiliations:** Nottingham Clinical Trials Unit, Nottingham Health Science Partners, University of Nottingham, Nottingham, UK; Inter-Disciplinary Ethics Applied, University of Leeds, Leeds, UK; Social Science Research Unit and EPPI-Centre, Institute of Education, University of London, London, UK; National Childbirth Trust, London, UK; Leeds Institute of Health Sciences, University of Leeds, Leeds, UK

**Keywords:** Research, Ethics, Pre-term/sick neonates, Parents, Clinicians, Consent, Validity, Competence, Voluntariness, Information

## Abstract

**Background:**

Conducting clinical trials with pre-term or sick infants is important if care for this population is to be underpinned by sound evidence. Yet approaching parents at this difficult time raises challenges for the obtaining of valid informed consent to such research. This study asked: what light does the empirical literature cast on an ethically defensible approach to the obtaining of informed consent in perinatal clinical trials?

**Methods:**

A systematic search identified 49 studies. Analysis began by applying philosophical frameworks which were then refined in light of the concepts emerging from empirical studies to present a coherent picture of a broad literature.

**Results:**

Between them, studies addressed the attitudes of both parents and clinicians concerning consent in neonatal trials; the validity of the consent process in the neonatal research context; and different possible methods of obtaining consent.

**Conclusions:**

Despite a variety of opinions among parents and clinicians there is a strongly and widely held view that it is important that parents do give or decline consent for neonatal participation in trials. However, none of the range of existing consent processes reviewed by the research is satisfactory.

A significant gap is evaluation of the widespread practice of emergency ‘assent’, in which parents assent or refuse their baby’s participation as best they can during the emergency and later give full consent to ongoing participation and follow-up. Emergency assent has not been evaluated for its acceptability, how such a process would deal with bad outcomes such as neonatal death between assent and consent, or the extent to which late parental refusal might bias results.

This review of a large number of empirical papers, while not making fundamental changes, has refined and developed the conceptual framework from philosophy for examining informed consent in this context.

**Electronic supplementary material:**

The online version of this article (doi:10.1186/s13063-015-0957-x) contains supplementary material, which is available to authorized users.

## Background

The recruitment of pre-term or sick infants to clinical trials requires approaching parents at a particularly difficult time, often within a tight timescale for making a decision. This raises challenges for the obtaining of valid informed consent to such research. But given the importance that has been attached by codes of ethics to gaining informed consent for medical research, if the problem of consent cannot be successfully addressed, there is a risk of this becoming an ‘orphan’ area of research.

This paper reports on research examining and addressing these difficulties. We undertook this systematic review of the empirical studies on these ethical challenges within the context of a larger project focused on improving care for the pre-term infant (with a special focus on the timing of cord-clamping).^1^

This is the first such review focusing on the ethical issues around consent to neonatal research. There have been other reviews which have focused on methods of increasing recruitment or of improving the means of conveying information, for example, but none that have focused directly on the ethical issues [1–6]. Our overall aim in conducting the review is to identify a way of mounting ethical neonatal research in those circumstances where obtaining valid consent from parents has proved a significant challenge. This is important since if research cannot be conducted ethically it will not be possible to provide a research service for this population.

Thus, this review aims to synthesise observational and qualitative studies that explore the process of consent to such trials, and report parents’ and clinicians’ views and experiences. The goal is to identify both the challenges to an ethically defensible consent process, and potential solutions, in order that ethical research can continue to be mounted in this area.

This approach to addressing problems in medical ethics is methodologically rather novel. In this case the research has been undertaken by a multi-disciplinary team with background expertise in philosophy, clinical practice, social science, information science and advocacy for parents, all of whom have brought complementary skills to the task. For the philosophers, the methods involved in systematic review were somewhat different from those that are conventionally adopted in work on ethics. The notion of a systematic review is very uncommon, indeed almost unknown, as a method for conducting research in philosophy, both in philosophy as a whole and in philosophical medical ethics in particular [7–9].

On the other hand, for the social scientists and clinicians the standard philosophical means for resolving ethical problems were unconventional by the standards of their disciplines. As a result the whole process of developing a systematic review in ethics through inter-disciplinary collaboration has given rise to considerable reflection in its own right. These unpublished observations of authors of this paper are being collated. However, for the purposes of this paper the method used in developing a systematic review will be described without significant space being given to detailed further reflection on that process.

The paper begins by setting out the method by which this systematic review was undertaken, then gives an account of the results, and concludes with a discussion of some of the key points arising from the results.

## Methods/Design

Our overarching question was: what light does the empirical literature cast on an ethically defensible approach to the obtaining of informed consent in perinatal clinical trials?

In broad outline the method adopted for this review conformed to that set out for a framework synthesis in Gough, Oliver and Thomas’s recent account of systematic reviews [10]. The first stage was the development of a tentative initial conceptual framework^2^ which relied mainly on prior knowledge of the existing philosophical literature on informed consent (prominent books and papers). This included knowledge of material that was more specifically focused on the difficulties of gaining informed consent in neonatal trials (in particular the Euricon study) [11, 12]. This initial conceptual framework informed the criteria for including studies.

An initial search of two databases of research literature (one medical and one philosophical) was then undertaken by an information specialist based on this tentative conceptual framework. The framework was then refined in light of the literature uncovered by the initial searches (stage 2 in Gough, Oliver and Thomas’ account, [10]), and the search strategy amended accordingly before being applied in full to a broader range of databases. At stage 3 the task of coding articles was undertaken, and this led to stage 4, the tabulating of the data. The final stage was then drawing conclusions from the tabulated data (stage 5).

### Methods

The aim of the search was to identify studies for a review of ethical issues around consent that arise from the involvement of either pre-term babies or sick neonates in clinical trials.

Articles potentially eligible for inclusion were those:

i). reporting any empirical research or analysis/commentary concerning;
  Consent, participation or recruitment for neonatal research (relevant to clinical trials)
  Parental decision-making for treatment of, or research with, sick or pre-term neonates
  Parental decision making for birth/labour
  Methodology in emergency/urgent neonatal research
  Alternative ways of gaining consent for neonatal research
ii). with full text available in English

As this focus raises issues that have previously been studied by multiple academic disciplines (e.g. obstetrics, fetal-maternal medicine, neonatology, social science, bioethics) a range of database sources were searched (see Additional file 1). Briefly, literature emerging from the draft searches using MEDLINE and Philosopher’s Index helped to develop the tentative conceptual framework further.

Titles and abstracts, and subsequently full texts, were retained only if they matched the inclusion criteria. Articles were screened by a researcher with clinical and ethics expertise (EW) who adopted an over-inclusive approach to avoid losing any relevant studies; when in any doubt the decision was discussed with a philosopher (CM).

The ‘included’ articles were then separated into ‘empirical’ (quantitative or qualitative research) and ‘analytical’ (philosophical discussion or commentary). The ‘empirical’ papers which were identified as potentially relevant after the screening process were re-read to identify recurrent themes. The themes that emerged were:

i). Attitudes of clinicians to parental consent
ii). Assessing the quality of consent
iii). Different methods of seeking and gaining consent
iv). Attitudes of parents and clinicians to neonatal trials
v). Best interests versus consent
vi). Antenatal counselling practices
vii). Clinical uncertainty
viii). Information transmission
ix). Perception of who has made the decision

Draft summaries of each theme were distributed to the steering group which comprised a social scientist, another clinician, a parents’ representative and the information specialist. After discussion with the steering group, the conceptual framework was modified to focus on the specific questions prioritised by the steering group. As a result, more focussed inclusion criteria were then set for the empirical papers, based on the modified conceptual framework. Articles finally eligible for inclusion were those:

i). reporting any empirical research or analysis/commentary concerning:
  Parents’ views of neonatal trials
  Clinicians’ views of neonatal trials
  Parents’ and clinicians’ views of parental consent/decision-making in clinical practice IF the articles concerned the validity of the consent in an emergency situation or during or soon after labour
  Validity of consent
  Other options for gaining consent
ii). with full text available in English

Further searches were devised with attention to the amended framework, terms suggested by the project team, database index terms, and recent literature listing terms useful in retrieving bioethics studies [8, 9, 13]. Additional file 1 shows an example search strategy. Additional databases were chosen to search based on their availability and the advice offered in the literature on bioethics studies retrieval, [8, 9, 13, 14].

The results of the further searches were then considered. Papers from these references were screened, and ‘included’ or ‘excluded’, based on abstracts using the second set of five inclusion criteria set out above. However, on this second screening addressing a focused review question allowed the review team to apply the concept of data saturation to the task of seeking additional studies [15] and demanded, in addition, very definite positive reasons to include further papers. There had to be clear indications that some new insights were being added to those we already had from the initial set of papers. In effect the approach here was analogous to treating research findings as saturated. Update searches were conducted on all previously chosen databases and subject to the same screening methods.

## Results and discussion

The PRISMA diagram (Additional file 2) illustrates the flow of studies throughout the review. The searches identified 1361 records (234 from the first searches, 790 from the second round, 327 from the update searches). A set of 41 papers identified from MEDLINE and Philosopher’s Index met the original inclusion criteria. Expanding the databases and introducing new search terms while narrowing the scope led to a total of 49 empirical papers being ‘included’.

Table 1 sets out the research context for these included studies.

**Table 1:** The context for the included studies

Context of study	Reviews	Questionnaires/Surveys	In-depth interviews	Miscellaneous
Emergency perinatal research	Hundley 2004 [56]	East 2006 [49]	Baker 2005 [24]	
	Tooher 2008 [30]	Kenyon 2004 [50]	Kenyon 2004 [50]	
		Smyth 2009 [22]	Smyth 2012 [23]	
			Snowdon 2006 [28]	
Emergency neonatal research		Ballard 2004 [46]	Allmark 2006 [47]	Schmidt 1999 (observation of outcomes) [57]
		Burgess 2003 [25]	Mason 2000 [34]	
		Culbert 2005 [53]	Snowdon 1997 [48]	
		Maayan-Metzger 2008 [19]	Snowdon 1998 [33]	
		Stenson 2004 [35]		
		Zupancic 1997 [21]		
Research during pregnancy		Daniels 2006 [52]	Mohanna 1999 [58]	
		Woodward 2012 [54]		
Non-emergency neonatal research		Ballard 2011 [51]	Hoehn 2009 [32]	Hoehn 2005 (analysis of unsolicited comments) [17]
		Burgess 2003 [25]	Jollye 2009 [18]	Hulst 2005 (observational study) [31]
		Hayman 2001 [16]	Korotchikova 2010 [29]	
		Marc-Aurele 2012 [45]	Rogers 1998 [55]	
		Morley 2005 [20]	Simon 2006 [60]	
		Nathan 2010 [59]	Snowdon 1998 [33]	
		Singhal 2002 [26]	Snowdon 2004 [42]	
		Singhal 2004 [41]	Snowdon 2004 [39] Ward 2009 [27]	
Clinical decision-making (not research)		Paulmichl 2011 [43]	Albersheim 2010 [40]	Sharma 2011 (analysis of decisions made by mothers) [64]
			Brinchmann 2002 [36]	
			De Leeuw 2000 [61]	
			Garel 2004 [62]	
			Kavanaugh 2005 [37]	
			McHaffie 2001 [38]	
			Saigal 1999 [63]	

The next stage (stage 3) was for the findings of these included empirical papers to be coded according to themes generated from the modified conceptual framework.

Table 2 presents the themes covered in this coding.

**Table 2:** The themes covered in the coding of the included papers

Themes covered	Reviews	Questionnaires/surveys	In-depth interviews	Miscellaneous
Attitudes of parents	Tooher 2008 [30]	Burgess 2003 [25]	Baker 2005 [24]	Hoehn 2005 [17]
		Hayman 2001 [16]	Brinchmann 2002 [36]	
		Maayan-Metzger 2008 [19]	Hoehn 2009 [32]	
		Morley 2005 [20]	Hulst 2005 [31]	
		Paulmichl 2011 [43]	Jollye 2009 [18]	
		Singhal 2002 [26]	Kavanaugh 2005 [37]	
		Smyth 2009 [22]	Kenyon 2004 [50]	
		Stenson 2004 [35]	Korotchikova 2010 [29],	
		Zupancic 1997 [21]	Mason 2000 [34]	
			McHaffie 2001 [38]	
			Smyth 2012 [23]	
			Snowdon 1998 [33]	
			Snowdon 2004 [39]	
			Snowdon 2006 [28]	
			Ward 2009 [27]	
Attitudes of clinicians		Paulmichl 2011 [43]	Albersheim 2010 [40]	
		Singhal 2004 [41]	De Leeuw 2000 [61]	
			Garel 2004 [62]	
			Garel 2011 [44]	
			McHaffie 2001 [38]	
			Snowdon 2004 [42]	
Validity of consent	Tooher 2008 [30]	Ballard 2004 [46]	Allmark 2006 [47]	
		Ballard 2011 [51]	Hoehn 2005 [17]	
		Burgess 2003 [25]	Hoehn 2009 [32]	
		Daniels 2006 [52]	Jollye 2009 [18]	
		East 2006 [49]	Kenyon 2004 [50]	
		Hayman 2001 [16]	Mason 2000 [34]	
		Kenyon 2004 [50]	Smyth 2012 [23]	
		Marc-Aurele 2012 [45]	Snowdon 1997 [48]	
		Nathan 2010 [59]	Snowdon 2006 [28]	
		Smyth 2009 [22]	Ward 2009 [27]	
		Stenson 2004 [35]		
Different consent processes	Hundley 2004 [56]	Ballard 2004 [46]	Allmark 2006 [47]	
		Ballard 2011 [51]	Hoehn 2009 [32]	
		Culbert 2005 [53]	Mohanna 1999 [58]	
		East 2006 [49]	Rogers 1998 [55]	
		Woodward 2012 [54]	Smyth 2012 [23]	
Miscellaneous topics			Rogers 1998 [55]	Schmidt 1999 [57]
			Saigal 1999 [63]	Sharma 2011 [64]
			Simon 2006 [60]	
			Snowdon 1998 [33]	

Once the papers had been coded, the results of that coding made it possible to tabulate the coded papers against the themes (tabulation of the results, stage 4). In effect this tabulation provides a summative identification of the key ethical points made in the papers analysed. The full findings are presented in Additional file 3. The findings discussed in this ‘results’ section are those which contributed to the broader theme particularly relevant to our research question.

In what follows, first of all we will present these tabulated results in prose form, and then in the last section we will discuss the conclusions to be drawn concerning the ethically significant features of those results. It should be noted that in tabulating the results we did not attempt to ‘weight’ the contribution of a paper to a theme according to the size or quality of the study, though the social scientist evaluated the papers and found them all of sufficient quality to be included.

Additional file 4 presents a table of all the empirical studies included in the review and their quality evaluation. All of the studies included at this stage met the quality assessment criteria.^3^

- *Attitudes of parents concerning consent in neonatal trials*

The empirical papers which explored ‘the attitudes of parents’ to consent in the process of research involving neonates covered a wide range of considerations of interest from the point of view of our research question, in particular: the motivations parents have for agreeing to research participation, or for declining; and their views and feelings about the research itself.

### Motivations of parents for consenting or declining to participate in research

There is consistency across multiple studies regarding the stated motivations for parents to consent or decline to participate in perinatal or neonatal trials. In favour of participation there was, on the one hand, ‘altruistic’ motivation^4^ – the benefit that entering the trial might bring to other babies, parents, doctors, or to society/science [16–24]. On the other hand, parents were motivated by the possibility of some benefit to their baby himself^5^ [17, 20, 25–27, 21, 23], or to the mother [23] – or the trial’s bringing some hope in a hopeless situation [28].

By contrast, parents also reported their motivations for declining to participate in research. There was the inconvenience for the parents [16, 29, 23, 30], but also the burden for their child [31, 24], whilst another consideration was worries about the risks involved, with concerns about the risks to their baby dominating those about risk to the mother [16–19, 26, 21, 22, 24, 30]. Finally, some felt that they did not have enough time to decide [32, 18]. However, it is reported that the severity of the illness of the infant did not affect trial participation [31, 18].

### Parental emotions regarding child’s participation in research

There are also a number of consistently reported feelings or emotions from parents about the medical/emergency context making them eligible to be approached for research. Thus, there were experiences of fear or dread [18, 28, 27, 23, 30], of confusion [18, 27, 23, 28], and of vulnerability [27, 24]. Other emotional responses were indirectly relevant to parental decision-making in that they reflected parental feelings after the decision had been made. These included: pride in having participated in the trial [18, 33, 23, 24]; although guilt after enrolling their child was also reported [18]. Another facet covered by the empirical research is the attitudes that parents take to the whole process of decision-making or consenting for trials. There are a number of findings here.

### Parental views on the process of deciding to consent or decline

First, parents feel that a formal consent form is necessary for research, and that this is necessary in order to protect their child [25]. Most parents also feel they ought to make the decision about whether their child participates in research [25, 34, 20, 26, 35], and reported reasons for this are similar across several studies. Thus, parents want to feel involved or informed about their child, and to maintain some control over what happens to him [36, 37, 28, 27]. A second reason is that it is part of the parents’ role or responsibility to take decisions of this sort [28, 27]. And this second reason is itself then grounded either in the unique relationship that parents have with their child [36] or in the fact that the parents must live with the outcomes of the decision [36]. Parents also feel they ought to take the decision in order to protect their baby from risk [18, 26, 24].

However many parents also wanted input from others as well before making the decision. This might include support from their spouse or wider family [32, 18, 29, 28, 23]. But they also noted the importance of a discussion with their doctor [32, 38, 28], either because they felt they lacked sufficient skills or knowledge to make the decision on their own [36], or because they put their trust in doctors to recommend the right decision [18, 23]. On the other hand some parents did not want to make the decision [36, 18, 21, 24].

In this connection there was a frequent finding that parents felt that being approached about a trial added to their stress and anxiety at a difficult time [36, 18, 35, 21, 23, 24], although this was not universal; two studies found parents reporting no extra burden [39, 35]. Parents also reported that the burden of decision-making increased if they were approached at an inappropriate time [23, 22, 24, 30].^6^

b) *Attitudes of clinicians concerning consent in neonatal trials*

Empirical research has also examined the attitudes of clinicians to the consent process and these studies make for an interesting comparison with the work on parents’ attitudes, which we will comment on in the concluding discussion. The views of clinicians covered a range of topics which overlapped with those on which parents commented but also covered issues going beyond those discussed by parents.

A major area of overlap concerns the attitudes that clinicians take to parental participation in the process of decision-making, or in consenting for research.

### Clinician views concerning parental participation in the consent process

Clinicians reported they respect parental authority [40] and for the most part clinicians feel that parental informed consent is necessary for trials [41, 42]. In justifying this, clinicians give a number of reasons for allowing parents to make the decision. First, there is respect for ‘parental rights’ [40] which might be seen simply as an elaboration of the notion of respect for parental authority. Then there is the claim that parents are best placed to act in the best interests of their child [40, 43, 41], and finally, clinicians note that the parents must live with the long-term outcomes of their decisions [40].

On the other hand some clinicians were more sceptical about the overriding importance of respecting parental authority. Thus some felt that clinicians are the best decision-makers for sick babies [43]. Along the same lines, in some studies clinicians reported prioritising their own idea of infant best interests over parental autonomy [40].

Some clinicians reached a closely related conclusion in virtue of wanting to spare parents the burden of making decisions [38, 44]. Similarly some reported involving parents by trying to ‘convince’ them of the need to consent [44]. However, some clinicians feel decisions should be made jointly [40]. In this context of reflecting on the role of clinicians and parents in decision-making, one study reported clinicians as seeing a conflict between infant best interests as a basis for parental decision-making and infant best interests as a limitation to parental authority [40].

### Clinician views on the effect of seeking consent on trial participation

Some clinicians are concerned that trial participation is dropping due to problems with obtaining consent [42]. On the other hand there was equal concern that parents could be pressured to participate [42] .

### Clinician views on the consent process

Studies also reported clinicians’ views on consent forms and communication. Thus, clinicians suggested two possibilities as to reasons for having consent forms and: that the forms are provided in order to protect researchers (by providing confirmation that information was given); and that they are there to aid communication with parents and improve their understanding [42]. However, clinicians also worried that too much information adds to the burden for parents who are already facing difficult circumstance [42]. In the same vein clinicians noted barriers to effective communication; namely, intimidation of parents [43], lack of care and support for them [43], failing to keep promises or raising false hopes [43], and caregivers presenting parents with diverging propositions about the research [43].

Finally, the empirical studies have reported on clinician views in two further slightly more theoretical areas concerning consent for research. Clinicians raised concerns about balancing their responsibility to the trial they were seeking to conduct with their responsibilities to the parents. Some took the view that these were ‘equal responsibilities’ – and that it is possible to discharge both responsibilities satisfactorily [42]. Others saw the possibility of ‘divided responsibilities’ – a possible conflict of interests, causing anxiety to clinicians [42] or of the need for ‘prioritised responsibility’ – in which clinicians must put parents’ interests before the interest of the trial [42].

c) *The validity of the consent process in the neonatal research context*

Mason and Allmark found that only 59 % of parents giving consent for neonatal trials had given valid consent according to self-reported problems with consent, in terms of either voluntariness, competence, or informedness (grasp of relevant information). This was based on a thematic analysis of interviews, and a subgroup analysis showed that this problem was even greater when parents were giving consent for urgent or emergency research [34].^7^

The analysis of the empirical papers revealed further views about the voluntary or coerced nature of the consent process, the extent to which parents were informed, and their competence or capacity to understand the information.^8^

#### i) Voluntariness and coercion or pressure

Some parents reported feeling pressure to participate [25, 16, 22], but others reported feeling no pressure [25, 17, 35]. Some parents knew they had the right to refuse to participate [25, 22, 45], but some did not [46]. Some demonstrated voluntary consent (according to some specific measure by the researcher) [47].

#### ii) Competence or capacity, and understanding

The competence, or capacity, of parents to give valid consent is potentially affected by a number of factors including emotional state, degree of understanding achieved and time available to decide. Studies covered all these factors. In terms of their emotional condition, in one study some parents reported they were calm when they made the decision [25], but others felt they had been anxious or stressed [25]. In some studies parents reported not making a proper decision [48, 28] due to pain [22], or due to anxiety and desperation. However, in others parents reported making considered and active decisions [25] despite pain [27], or time pressure [32] or anxiety and desperation [47, 28].

So far as understanding was concerned, some parents reported clear understanding of a trial [25, 48, 35, 49] and some parents demonstrated clear understanding of a trial (according to some specific measure) [18, 45, 47]. However, some parents reported problems with understanding a trial about which they were asked for consent [32, 34, 23, 22], and some parents demonstrated little or no understanding of such a trial (according to some specific measure) [46, 25, 50]. Some parents felt they could isolate a ‘critical factor’ to make a genuine decision even with suboptimal understanding [28]. Some parents showed that they had not understood risks correctly (when compared to researchers’ specific classification of risks) [46, 51, 28]. Some showed deficiency in understanding trial methodology [30, 45] and it was reported that the communication skills of the clinician affects understanding, and could sometimes be deficient in some ways [43, 23].

Time might be a factor which can affect capacity or competence in a particular situation because of its bearing on the ability, in the circumstances, to process information. Two studies (each concerning more than one trial with different timescales and different levels of risk) found that while the majority of parents felt they had adequate time, in the circumstances, to make the decision a significant minority did not [25, 32]. In this context, some studies found that parents made rapid decisions regardless of how much time was actually available [28]. On the other hand, others found parents needed more time the greater the risk [28]. Furthermore, other studies found that with non-urgent trials there was a gradual understanding and acceptance of information over time [18].

#### iii) Information

In order to give valid consent, parents must receive the relevant information. Some parents reported receiving satisfactory information [25, 35, 52, 49, 45], and some parents demonstrated having satisfactory information (according to some specific measure) [47]. However, some parents reported problems with the information they were given, or an unsatisfactory level of information [34, 22]. In particular the information sheet is intended to play a significant role here, but was found often to be unread [34]. But in some studies parents remembered being given an information sheet [18, 35, 23, 50], and parents used the information sheet when making the decision [18, 23 (less than half), 50]. With respect to another important consideration, some parents reported having no opportunity to ask questions [25], but other parents in the same study remembered having the opportunity to ask questions [25].

d) *Different methods of obtaining consent*

Empirical studies have explored parental attitudes (but not, it seems, clinician attitudes) to various methods of obtaining valid consent which have been proposed in the literature or trialled. In general, mothers acknowledge the difficulties for researchers in finding the ‘right time’ to approach them for consent for perinatal research [23].

#### i) Antenatal consent

It was found that some parents would prefer antenatal consent rather than consent during, or after, labour [53, 32, 54, 23], and it was found that parents would like the information earlier in pregnancy even if they were not recruited then. However, it was also found that parents were not completely comfortable with antenatal consent [53, 32]. Thus, parents reported not seeing the relevance of the trial at the time of antenatal consent – ‘it will never happen to me’, and also reported increased burden or anxiety if told about the trial earlier in pregnancy [23].

#### ii) Consent in labour

Iit was found that some parents were comfortable with consent in labour [23], although this is a burden on staff at a busy time [49], but that some parents were not comfortable with consent in labour [53, 23].

#### iii) Waived consent

It was found that parents were not comfortable with waived consent [53].

#### iv) Opting out

In this method consent is presumed unless the parent explicitly opts out of the trial as opposed to conventional consent when the parent has to positively choose to enter the child (and this was examined for a case when both were done antenatally): it was found that some parents were comfortable with opting out [55], but also that some parents were not comfortable with opting out [53]. In addition, more parents were recruited via the opt-out method [55] and understanding was reported as better in opt-out consent [55], but the opt-out consent method did not reduce the burden on parents [55].

#### v) Continuous consent

In this approach, advocated by Allmark and Mason [40], there is initial agreement to participate, but then continuing discussion and further information after recruitment.) It was found that the validity of the consent improves when discussion continues after recruitment (presumably this refers to the validity of the later ‘continued’ consent not of the original consent, whose validity cannot be changed by later events) [47, 46]. Some parents approve of continuous consent [47], but some parents have concerns about continuous consent – concerns about receiving further information at a later stage when that might have affected their original decision [47].

#### vi) Staged consent process

An empirical study has reported on a staged consent process in which consent (oral or written) is sought antenatally, and the consent is sought again at the point of intervention. However, at present this is only reported not discussed [56].^9^

#### vii) Enhanced consent

Where parents received standard counselling plus additional material including a one-page summary of the trial and a set of frequently asked questions, this was found (by researchers) to result in better understanding than conventional consent, but still to leave significant gaps in understanding for most parents [51].

## Conclusions^10^

Our main aim in conducting this review has been to establish an ethically acceptable way for conducting neonatal research. In that context the main conclusions are that:

1. There is widely held agreement that it is important that parents do give or decline consent for neonatal participation in trials; but that
2. There is evidence that existing consent procedures are unsatisfactory; and that
3. None of the proposed alternative consent processes reviewed by the research is satisfactory; and
4. There are some significant gaps in the empirical research in this area.

### Parental and clinician attitudes

#### Agreement on the need for parental consent

A key conclusion is that although parents and clinicians are quite divided about a number of issues regarding the consent process, there is almost universal agreement about the importance of parents giving (or declining) consent for neonatal participation. This is very important because one possible response to the difficulty of conducting neonatal research ethically is to suggest that in this area the requirement for informed consent should be dropped. This research strongly rebuts that proposal.

Despite the variety of views, the studies reveal that parents very generally see it as important that parents give consent to (or decline) their child’s participation in trials, and they have several good reasons for this.^11^ However, they may not be fully aware of the challenges to giving valid consent to research. Likewise, clinicians too do have reasons for respecting parental authority (though not always for exactly the same reasons, and it is interesting what reasons they agree upon and that they disagree about, according to the data).

Furthermore, clinicians, as well as parents, can view the problem not just in terms of consent but in terms of the child’s best interests, and in terms of the value of research. And although parents generally wish to make the decision, often they desire advice from doctors, recognising their skills and experience.

The results also show how, in the complexities of actual practice, the very same events in a process can both be seen in different ways (as the data suggests) and slide from being one kind of act into being a different kind. So seeking advice (parental view) and giving advice to inform a decision (clinical view) might be, or become, seeking direction (parental view) and giving a very definite steer (clinical view), which might be, or become, the parent not wishing to decide (parental view) and the clinician relieving the parent of the ‘burden’ of decision- making (clinician view).

This last point might be taken as evidence that seeking parental consent is not necessary. But, to reiterate our conclusion, the results show clearly that there is wide agreement (amongst most parents and clinicians) that it is important that parents do give or decline consent for neonatal participation. And thus there is a need to find a suitable process.

#### Divergent parental and clinician perspectives

Clearly parents and clinicians approach the issues examined in empirical research from different perspectives: on the one hand (parents) as potential or actual givers of consent (or decliners) for participation in a neonatal trial; on the other hand (clinicians) as researchers responsible for delivering important research, but often also as carers with responsibility for the best interests of the neonate (and possibly also the mother, in the case of fetal-maternal research). This means that the focus of empirical research covers some questions which only really apply from the parental perspective, but others which apply only from the perspective of a clinician/researcher. However, there is a third area where questions addressed to clinicians and parents intersect.

#### Difficulties in giving valid informed consent

Research which focused primarily on parents covered matters such as their emotional state and their understanding of information and, although views were not uniform, confirmed that there are difficulties for at least some parents in both areas, from the point of view of giving valid consent. With respect to the clinical perspective the studies revealed both clinical concerns that neonatal research participation is dropping due to problems with consent, and also the ethical difficulties that clinicians face in discharging conflicting duties to research, neonates, and parents.

Perhaps the most interesting area covered by the studies is that where parental and clinical responses intersect, for the results here bring out forcefully the rather messy nature of practical attempts to address the ethical issues around consent for research in circumstances (such as perinatal) where gaining valid consent is difficult. This is both reflected in and partly explains the variety of views represented in the research. And one might add that where there are clashes in view, that might also be partly explained by the fact the parents tend to see these situations from a) the perspective of parental responsibility to children, and b) their limited experience of the challenges in giving valid consent in these circumstances, whilst clinicians naturally also view it from c) the point of view of a clinical responsibility for patient interests, but also d) that of a researcher worried about the challenge of getting real consent and e) that of a researcher concerned to take forward good research in this area and to avoid it becoming an ‘orphan’ research area.

### Validity of consent and modes of consent process

The second main conclusion is that existing consent processes do not lead reliably to valid parental consent, and the third that there is an urgent need to find an effective procedure.

#### Parents fail to give valid consent

Although there is a division of parental opinion, the studies show conclusively that enough parents have failed to give valid consent (measured against one or other of the three criteria of validity – voluntariness, informedness and competence) to suggest that concerns about current efforts to obtain parental consent to neonatal participation in perinatal research are warranted.

#### Alternative methods of gaining consent are not satisfactory

Our third concluding point coheres with this. It is that the studies discussed in the fourth section of results above confirm that, even on empirical grounds, none of the varied processes adopted in order to obtain valid informed consent in neonatal trials are working sufficiently well. Although some parents are ‘comfortable’ with each method, other parents are not. It is very likely that this is because, for all the methods considered, valid consent is not being achieved for some parents.^12^

Taking these three main points together, it follows, then, that there is that a need for a suitable consent process for neonatal trials, but that none of the procedures examined in the empirical studies to date look to be adequate. So there is a need for another process.

Now, for urgent and emergency research there is in fact a distinct possible consent process, described below. However, our fourth conclusion is that the empirical studies do not as yet provide any data on attempts to implement this procedure. So we now turn to this important point.

### Gaps in the empirical literature

Although large, our search has nonetheless identified important gaps in the empirical literature relevant to consent for perinatal trials.

The first gap is the most important with respect to the goal of finding an ethically acceptable way to conduct neonatal research. This is the lack of any study on a new process for obtaining urgent or emergency consent in perinatal research where there is very little time in which to seek consent to participation. There do not seem to be any empirical studies of such a process. This process involves seeking ‘assent’ to participation in the trial from parents at the outset, accepting that there is insufficient information for fully-informed consent at that point, but then providing further information over time which allows the parents to give full consent (or an informed withdrawal), which is different in kind from the assent sought at the outset.^13^ Such a process may make ethically acceptable neonatal research possible in urgent/emergency situations so empirical work on its effectiveness is a significant gap to fill.

A second gap concerns empirical studies on fetal-maternal research, that is trials in which both the mother and the fetus or neonate are participants and thus in which both fetal/neonatal interests and maternal interests are in question. Our review did not uncover studies that considered consent for research in this context.

Finally, a third gap concerns empirical studies which report the views of bereaved parents on the consent process. Up till now empirical studies have almost completely excluded views of parents whose sick child participated in research and survived.^14^

## Additional files

Additional file 1:
**Full tabulated findings.** Full findings from all the papers included. (The findings discussed in the results section are those which contributed to the broader theme particularly relevant to our research question). (PDF 134 kb) Additional file 2:
**Search strategy.** Databases searched and example search strategy. (PDF 88 kb) Additional file 3:
**PRISMA flow diagram.** Diagrammatic representation of the study flow right through the review process. (PDF 177 kb) Additional file 4:
**The evaluation of the quality of the empirical studies included in the review.** Diagrammatic presentation of the assessment of each study against the quality criteria. (PDF 92 kb)

## Abbreviations

NIHR: National Institute for Health Research
PRISMA: Preferred Reporting Items for Systematic Reviews and Meta-Analyses

## Footnotes

1 NIHR Programme in Applied Health Research, Improving quality of care and outcome at very preterm birth (RP-PG- 0609-10107).

2 In philosophical work this is sometimes arrived at using a mind-map technique.

3 As Additional file 4 shows only 3 studies included questions about hypothetical scenarios as opposed to actual consent processes, and 2 of these were with clinicians not parents.

4 Scare quotes are used here as, to the extent the parents are deciding whether or not to enter their child, it is not directly altruism on their behalf, since the child is the one entering the trial, not the parents. Of course in some cases, as in cord-clamping trials, it might be both mother and child that enter the trial. As will be discussed later, one gap in the empirical literature was study of cases of maternal-fetal research.

5 We follow the convention of referring to the baby as ‘him’ (using the ungendered form) to distinguish the baby from the mother in what follows.

6 Additional file 3 gives additional findings for this section and for further sections.

7 More recently Nathan found – using a competence assessment tool – that parents of sick neonates gave significantly more valid consent than patients with Alzheimer’s or schizophrenia, though comparison with previously published control groups using the same assessment tool gave variable results [58]. But this does not necessarily show that their consent was valid.

8 Voluntariness, informedness and competence (rationality) are the three standard criteria for informed consent.

9 The authors raise the question of whether the initial consent is really ‘permission to be approached later’ and the ‘actual consent’ for the study then takes place at the second discussion.

10 Full tabulated results are presented in Additional file 1.

11 Though not all the reasons are good ones. The consent process might be seen as having a very small role in protecting neonates from risk, but the main responsibility for that lies with research designers and research ethics committees which are much better placed than most lay parents to make such judgements of risk. This is pretty clear on philosophical grounds alone, but one piece of data in the studies (about parental assessment of risks) also supports this.

12 It might be suggested that with respect to these second and third claims the force of an appeal to empirical data alone is less compelling. This is because, although parental claims about how comfortable they felt in a consent process are of interest, it is very likely that parents in general will not have thought through the criteria for valid informed consent, so not appreciate fully the challenges to providing genuinely valid informed consent in certain neonatal research contexts. So even where a parent reports ‘comfort’ with a consent process this is not sufficient to show that such a process was a satisfactory means of gaining valid consent.

13 Allmark’s suggestion is similar to this, but his version is expressed in terms of continuous consent [46].

14 A review by Claire Snowden et al. on the BRACELET research has now reported on views of bereaved parents, but this research was published after the our review was completed. A reviewer notes that they were also considered by Allmark and Mason [46] .
