# Supplementary figures and images for: The ethical issues regarding consent to clinical trials with pre-term or sick neonates: a systematic review (framework synthesis) of the empirical research
Source: Trials. 2015 Nov 4;16:502. doi: 10.1186/s13063-015-0957-x (PMC4634156; doi:10.1186/s13063-015-0957-x)

## Additional file 2 PRISMA Flow Diagram

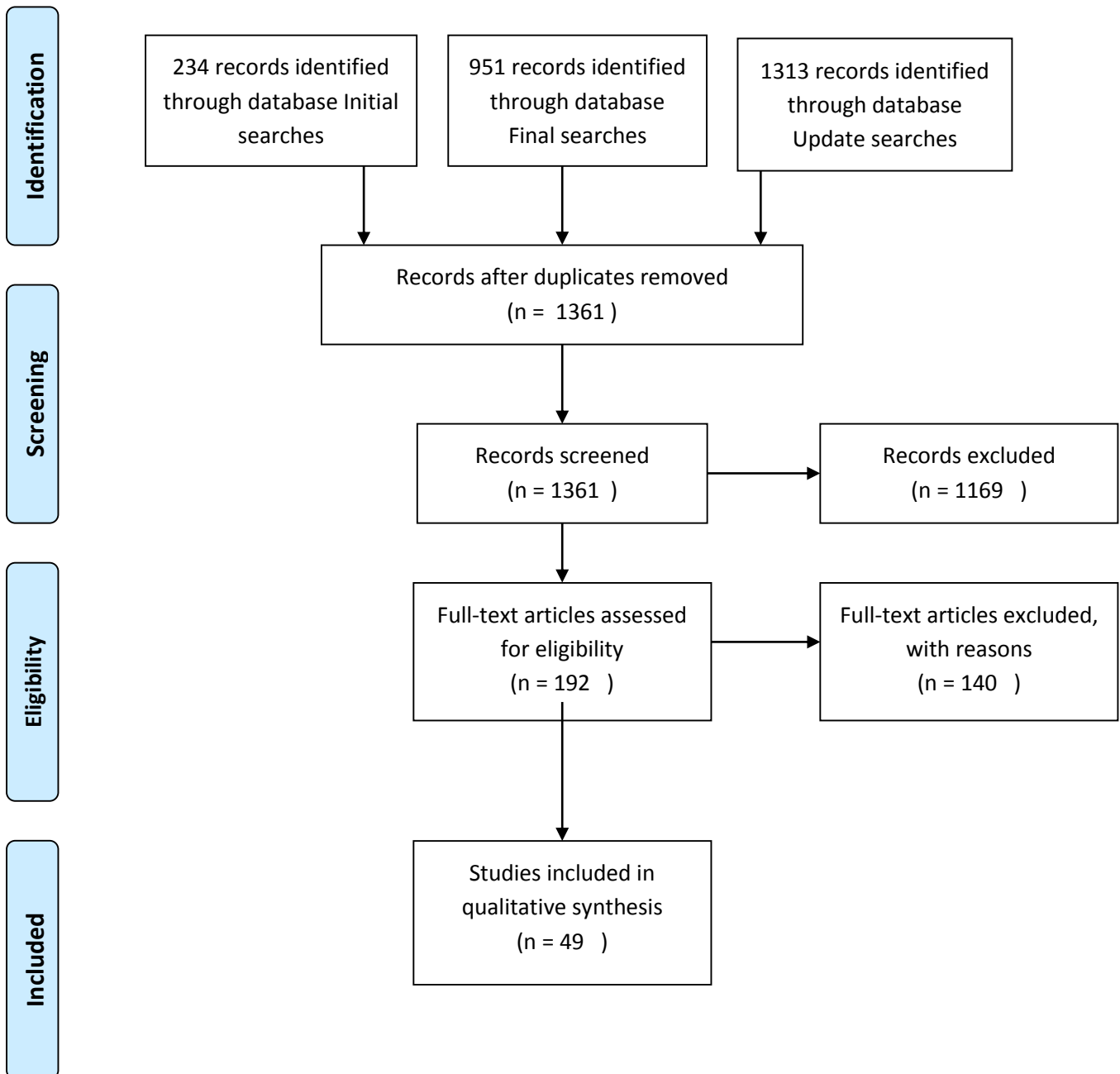

Supplement: Additional file 2: — Search strategy. Databases searched and example search strategy. (PDF 88 kb) [file 13063_2015_957_MOESM2_ESM.pdf]
