# Supplementary material for: The ethical issues regarding consent to clinical trials with pre-term or sick neonates: a systematic review (framework synthesis) of the empirical research
Source: Trials. 2015 Nov 4;16:502. doi: 10.1186/s13063-015-0957-x (PMC4634156; doi:10.1186/s13063-015-0957-x)
Supplement: Additional file 3: — PRISMA flow diagram. Diagrammatic representation of the study flow right through the review process. (PDF 177 kb) [file 13063_2015_957_MOESM3_ESM.pdf]

Additional File 3: Full tabulated findings

Full findings from all the papers included. (The findings discussed in the results section are those which contributed to the broader theme particularly relevant to our research question.)

ATTITUDES OF PARENTS

Attitudes to and feelings about research itself and motivations to participate/decline

- Parents described two main motivations to participate
    - 'altruism' – benefit to other babies/parents/doctors/society/science [16-24](Benefit to the baby [17, 20, 21, 23, 25-27]
        - Some hope in a hopeless situation [28]
        - Baby will get better care in a trial
    - Benefit to mother [23]
- Motivation to decline
    - Inconvenience for the parents [16, 23, 29, 30]
    - Burden for the child [24, 31]
    - Worries about risk [16-19, 21, 22, 24, 26, 30]
        - Concerns about risks to baby dominate concern about risk to mother [30]
    - Not enough time to decide [18, 32]
    - Loss of individualised approach from clinicians [24] Approached for too many trials [24]
- Severity of illness of the infant did not affect participation [18, 31]
- Reaction to encounter with the clinician/researcher affected decision to participate or not [28, 30]
- Recruitment process affected decision to participate or not [24]
- Feelings of parents about situation/illness of child
    - Fear/dread [18, 23, 27, 28, 30]
    - Confusion [18, 23, 27, 28]
    - Vulnerability [24, 27]
- Feelings about the trial
    - Guilt after enrolling [18]
    - Relief when trial over [18]
    - Concerns about randomisation [24]
        - What if they are in the wrong arm [18]
    - Pride in having participated [18, 23, 24, 33]
    - Disappointed if not approached [24]
    - Guilt after declining to participate[18]
    - Concern that decision about participation could affect standard of care [24]

Attitudes to decision-making and consent – research and clinical decisions

- Parents feel a formal consent form is necessary for research
    - To protect the child [25]
- Most parents feel they ought to make the decision [20, 25, 26, 34, 35]
    - To feel involved/informed/maintain some control [27, 28, 36, 37]
    - Because parents are the best decision-makers[43]
    - Because it is part of the parental role/responsibility [27, 28]

        - Due to the unique relationship with the child [36]
        - Because the parents must live with the outcomes [36]
    - To protect the baby from risk [18, 24, 26]
- But many wanted input from others too before making the decision
        - Support from spouse/family [18, 23, 28, 29, 32]
            - More likely to seek father's advice if affect on baby is anticipated [24]
            - Ultimate decision felt to be mother's alone if recruited during pregnancy [23]
        - Discussion with their doctor [28, 32, 38]
            - Because they feel they lack sufficient skills knowledge on their own [36]
            - Because they put their trust in doctors to recommend the right decision[18, 23]
        - When there was significant input and 'recommendation' from doctors, there was variation among parents in their perception of who had actually made the decision [37, 38]
- Time pressure
    - Some studies found that parents made rapid decisions regardless of how much time was actually available [28]
    - Others found parents needed more time the greater the risk [28]
    - Others found that with non-urgent trials there was a gradual understanding and acceptance of information over time [18]
- Some studies found parents felt being approached about research added to their stress and anxiety [18, 21, 23, 24, 35, 36]
- Other studies found no extra burden for parents when being asked about research [35, 39]
- Burden of decision-making increased if approached at 'inappropriate' time [22-24, 30]
- Some parents did not want to make the decision [18, 21, 24, 36]
- Parents idea of barriers to effective communication
    - Absent linguistic empathy [43]
    - Lack of concern for parents' interests and autonomy [43]
    - Use of abstract and vague terms [43]
    - Arrogant, irreverent or dismissive remarks [43]

ATTITUDES OF CLINICIANS

- In most studies clinicians report they respect parental authority [40]
    - Clinicians feel parental informed consent is necessary for trials [41, 42]
- But in some studies clinicians reported
    - or prioritising their own idea of infant best interests over parental autonomy [40]
    - sometimes demonstrating that they override parental decisions in their responses to scenarios even when they had reported respecting parental authority [40, 44, 61,62]
- Some clinicians feel decisions should be made jointly [40]
- Some report involving parents by trying to 'convince' them [44]
- Some want to spare parents the burden of making decisions [38, 44]
- Clinicians idea of the reason for allowing parents to make decision
    - 'parental rights' [40]
    - Parents best placed to act in best interests [40, 41, 43]
    - Parents must live with long-term outcomes [40]
- But some felt that clinicians are the best decision-makers for sick babies [43]
- One paper recognized a conflict between infant best interests as a basis for parental decision-making and infant best interests as a limitation to parental authority [40]
- Clinicians were concerned that research participation is dropping due to problems with consent [42]
    - Equal concern that parents could be pressured to participate [42]
- There was concern about balancing responsibility to trial with responsibility to parents
    - 'equal responsibility' – possible to discharge both responsibilities [42]
    - 'divided responsibility' – possible conflict of interests, caused anxiety to clinicians [42]
    - 'prioritised responsibility' – parents come before trials [42]
- Varied feelings as to whether it is
    - Appropriate [42]
    - Or inappropriate [42]
    - To approach bereaved parents to participate for 'altruistic' reasons.[42]
- Attitudes to formal consent forms
    - There to protect researchers [42]
    - There to aid communication/understanding [42]
    - Too much information adds to burden for parents [42]
- Clinicians views of barriers to effective communication
    - Intimidation [43]
    - Lack of care and support of parents [43]
    - Unkept promises and false hopes [43]
    - Diverging propositions between caregivers [43]

VALIDITY OF CONSENT

Background/general points

Mason and Allmark found that only 59% of parents giving consent for neonatal trials had given valid consent according to self-reported problems with consent from thematic analysis of interviews, and a subgroup analysis showed that this problem was even greater when parents were giving consent for urgent or emergency research [34]. Nathan found – using a competence assessment tool – that parents of sick neonates gave significantly more valid consent than patients with alzheimers or schizophrenia, though comparison with previously published control groups using the same assessment tool gave variable results [59].

Pressure/Coercion/Voluntariness and capacity

- In one study some parents reported they were calm when they made the decision [25]
- But others they felt they had been anxious or stressed [25]
- In some studies parents reported not making a proper decision [28, 48] due to
    - Time pressure [22]
    - Pain [22]
- In others parents reported making considered/active decisions [25] despite
    - Time pressure [32]
    - Pain [27]
    - Anxiety/desperation [28, 47]
- Some parents reported feeling pressure to participate [16, 22, 25]
- Others reported feeling no pressure [17, 25, 35]

- Some felt they had adequate time to make the decision [25]
- Some did not [25, 32]

- Some parents knew they had the right to refuse to participate [23, 25, 45]
- Some did not [46]

- Some demonstrated voluntary consent (according to some form of objective measure by the researcher) [47]

Understanding

- Some parents reported clear understanding of a trial [25, 35, 48, 49]
- Some parents demonstrated clear understanding of trial (according to some objective measure) [18, 45, 47,]
- Some parents reported problems with understanding [22, 23, 32, 34]
- Some parents demonstrated little/no understanding (according to some objective measure) [25, 46, 50]
- Some parents felt they could isolate a 'critical factor' to make a genuine decision even with suboptimal understanding [28]

- Some parents showed that they had not understood risks correctly (when compared to researchers classification of risks) [22, 28, 30, 46, 51]
- Deficiency in understanding trial methodology [30, 45]
- Communication skills of clinician affects understanding [23]

Information

- Some parents reported problems with the information they were given [34]
- Parents remembered being given an info sheet [18, 23, 35, 50]
- Parents used the info sheet when making the decision [18, 23](less than half of parents) [50]
- Parents reported having no opportunity to ask questions [25]
- Parents remember having the opportunity to ask questions [25]
- Parents reported satisfactory information [25, 35, 45, 49, 52]
- Parents demonstrated satisfactory information (according to some objective measure) [47]
- Parents report unsatisfactory level of information [22, 34]

Some studies showed that mothers' consent was valid less often/had more problems with validity than fathers' [16, 25, 47]

Others showed that mothers had fewer problems with understanding/information than fathers [46]

DIFFERENT METHODS OF GETTING CONSENT

- Antenatal consent
    - Parents would prefer/are happy with antenatal consent to consent during/after labour [23, 32, 53,54]
    - Parents not comfortable with antenatal consent [23]
    - Parents don't see relevance of trial at time of antenatal consent 'it will never happen to me' [23]
    - 'Non compliance' is common (recruited and randomised antenatally then not eligible at time of intervention – problem for trial methodology) [56]
    - Increased burden/anxiety if told about the trial earlier in pregnancy [23]
    - Parents would like 'info' earlier in pregnancy even if not recruited then [23, 49]
- Consent in labour
    - Parents not comfortable with consent in labour [23, 53]
    - Selection bias towards those presenting in early labour [49]
    - Burden on staff at a busy time [49]
- Waived consent
    - Parents not comfortable with waived consent [53]
- Opting out (compared to conventional consent when both done antenatally)
    - Parents comfortable with opting out [55]
    - Parents not comfortable with opting out [53]
    - More parents recruited via opt-out method [55]
    - Opt-out does not reduce the burden on parents [55]
    - Understanding is better in opt-out consent [55]
- Continuous consent (Allmark – continuing discussion and further information after recruitment)
    - Validity of consent improves when discussion continues after recruitment (presumably validity of continued consent not of original consent) [46, 47]
    - Parents approve of continuous consent [47]
        - Parents have concerns about continuous consent
        - Getting further information which might have affected original decision [47]
- Staged consent – consent (oral or written) antenatally, then repeated at point of intervention.
    - High drop-out rate as with antenatal consent [49]
- Enhanced consent (Ballard)
    - Results in better understanding [51]
- Women acknowledge the difficulties for researchers in finding the 'right time' to approach for perinatal research [23]

MISCELLANEOUS THEMES

- Parents are more accepting of severe disability states than health professionals [63]
- Adolescents' views of above are more consistent with their parents' than with health professionals [63]
- Sick newborns benefit from being in a trial even if in the placebo group[57]
- Clinical recommendations based on absolute risks can differ from patient priorities related to the same risks [64]
- Parents feel it is important to be given the results of a trial in which their child has participated [33]

- Better communication is open (no taboos), clarified (no vague or abstract terms) and uses defined language [43]
- Clinicians mention altruism when discussing trials with patients [60]
    - Discussing altruism has no effect on the recruitment rate [60]
    - Discussions of altruism involve
        - ‘others’ benefiting
            - science/knowledge [60]
            - other patients/children [60]
            - ‘the future’/’other people’ [60]
        - Normative judgements about participating in research (good and compassionate to participate, selfish and cowardly not to) [60]
