# Supplementary material for: The ethical issues regarding consent to clinical trials with pre-term or sick neonates: a systematic review (framework synthesis) of the empirical research
Source: Trials. 2015 Nov 4;16:502. doi: 10.1186/s13063-015-0957-x (PMC4634156; doi:10.1186/s13063-015-0957-x)
Supplement: Additional file 4: — The evaluation of the quality of the empirical studies included in the review. Diagrammatic presentation of the assessment of each study against the quality criteria. (PDF 92 kb) [file 13063_2015_957_MOESM4_ESM.pdf]

Additional file 4 : The Evaluation of the Quality of the Empirical Studies Included in the Review

	**Is it clear whose perspective is offered?**	**Are the data collection and analysis methods clearly described?**	**Were questions for data collection open or closed? Was the analysis grounded in the data or framed externally?**
Albersheim 2010 [40]	Yes, practicing neonatologists in Canada	Yes, semi-structured interviews and comments on scenarios<br>Yes, thematic analysis of interviews and quantitative analysis of responses to scenarios	Open and closed questions.<br>Externally framed analysis
Allmark 2006 [47]	Yes, parents who gave consent to the TOBY trial and clinicians who sought it, in UK	Yes, semi-structured interviews<br>Yes, framework analysis	Open questions<br>Flexibly framed analysis
Ballard 2004 [46]	Yes, parents of newborns enrolled to the NEOPAIN study in Kentucky, USA	Yes, open ended questions.<br>Yes, questions translated into a closed Likert score and analysed quantitatively	Open questions<br>Externally framed analysis
Ballard 2011 [51]	Yes, parents of newborns enrolled to a specific phase II study in the Neonatal Intensive Care Unit at the University of Kentucky, USA	Yes, open ended questions asked during a controlled trial comparing standard consent with enhanced consent.<br>Yes, questions translated into a closed Likert score and analysed quantitatively	Open questions<br>Externally framed analysis
Brinchmann 2002 [36]	Yes, parents who had experienced one or more life-and-death decisions relating to their critically ill and/or premature infant, in Norway	Yes, in-depth interviews<br>Yes, thematic analysis discussed in the light of theories of ethics	Open questions<br>Emergent themes framed by theories of ethics
Burgess 2003 [25]	Yes, parents who agreed, and those who refused, to enrol their newborns into trials in a NICU in a tertiary care referral centre, Canada	Yes, questionnaire with Likert scale and 3 open questions<br>Yes, quantitative analysis	Closed and open questions<br>Externally framed analysis
Culbert 2005 [53]	Yes, parents who had received obstetrical or neonatal care at a tertiary perinatal centre, Denmark	Yes, mailed questionnaire with Likert scale<br>Yes, quantitative analysis	Closed questions<br>Externally framed analysis

De Leeuw 2000 [61]	Yes, Physicians and nurses in 143 NICUs across Europe	Yes, self-administered Questionnaire<br>Yes, quantitative analysis	Closed questions<br>Externally framed analysis
Garel 2004 [62]	Yes, obstetricians and midwives in three tertiary-care maternity units, France	Yes, semi-structured interviews<br>Yes, content analysis	Open questions<br>Analysis grounded in the data
Garel 2011 [44]	Yes, physicians and nurses in thee NICUs, France	Yes, semi-structured interviews<br>Yes, content analysis	Open questions<br>Analysis grounded in the data
Hayman 2001 [16]	Yes, parents who consented or declined to participate in a study of SIDS, New Zealand	Yes, questionnaire<br>Yes, quantitative analysis	Closed questions<br>Externally framed analysis
Hoehn 2005 [17]	Yes, parents of neonates having cardiothoracic surgery, a Children's Hospital, Philadelphia, USA	Yes, Semi-structured qualitative interviews<br>Yes, thematic analysis of sponateously offered reasons for or against research participation	Open and closed questions<br>Themes emergent from the data
Hoehn 2009 [32]	Yes, parents of neonates with critical congenital heart disease, who had made decisions either for or against research participation before their neonate's cardiothoracic surgery, Chicago, USA	Yes, semi-structured interview<br>Yes, thematic analysis	Open and closed<br>Themes emergent from the data
Hulst 2005 [31]	Yes, parents or legal representatives of children (pre-term neonates to 18 years) in ICU who were approached for consent to enrol their child in a nutritional assessment study, The Netherlands	No<br>Yes, quantitative analysis	Closed questions<br>Externally framed analysis
Jollye 2009 [18]	Yes, parents who had chosen or declined to participate in clinical trials, England	Yes, semi-structured interviews<br>Yes, thematic analysis	Open questions<br>Themes emergent from the data
Kavanaugh 2005 [37]	Yes, mothers hospitalised for risk of pre-term birth; their partners, physicians and nurses, USA	Yes, serial interviews with parents; single interviews with each clinician.<br>Yes, analysed in line with Ottawa Decision Support Framework	Open questions<br>Externally framed analysis
Korotchikova 2010 [29]	Yes, parents of healthy full-term newborn babies on the postnatal wards in a University	Yes, observation of presence/ absence partner	Open questions<br>Externally framed analysis

	Maternity Hospital, Ireland	Yes, quantitative analysis according to four principles of the consent process	
Maayan-Metzger 2008 [19]	Yes, mothers of healthy term newborns and mothers of stable growing preterm infants with no active disease who had given birth at a large tertiary hospital, Israel	Yes, questionnaire with Likert scale<br>Yes, quantitative	Closed questions<br>Externally framed analysis
Mason 2000 [34]	Yes, parents who had been asked for consent to neonatal trials and neonatologists seeking consent, in Europe	Yes, semi-structured interviews<br>Yes, quantitative analysis according to four principles of the consent process	Open questions<br>Externally framed analysis
McHaffie 2001 [38]	Yes, doctors and nurses working in six large neonatal intensive care units (NICUs) throughout Scotland	Yes, in-depth, face-to-face interviews with semi-structured schedules<br>Unclear, 'Data were entered onto a computer under predetermined variable names, with scope for unlimited values to accommodate the full range of qualitative responses'	Open questions<br>Analysis unclear
Morley 2005 [20]	Yes, parents of premature babies in the neonatal intensive care unit, Australia	Yes, questionnaire with multiple choice answers or Likert scale<br>Yes, quantitative	Closed questions<br>Externally framed analysis
Nathan 2010 [59]	Yes, parents of neonates undergoing cardiac surgery who had made decisions about research participation before their neonate's surgery, USA	Yes, MacArthur competence assessment tool for clinical research (MacCAT-CR), specifically testing the components of understanding, appreciation, reasoning and choice<br>Yes, quantitative	Unclear whether questions open or closed<br>Externally framed analysis
Paulmichl 2011 [43]	Yes, clinicians involved in various dilemmas of neonatology (nurses, obstetricians, neuro-paediatricians and neonatologists, midwives and other health professionals such as	Yes, a questionnaire for professionals; for parents, similar questions asked in interviews no more than 30 minutes long	Some open, some closed<br>Content analysis and descriptive statistics for analysing answers to questions

	clinical psychologists and physical therapists); and parents of preterm and term infants treated in NICU		Unclear to what extent analysis externally framed
Rogers 1998 [55]	Yes, mothers of low birth weight infants eligible for an RCT of primary care follow-up, Texas, USA	Yes, structured interviews<br>Yes, RCT of conventional or modified consent procedure	Closed and open questions<br>Pre-set outcomes
Saigal 1999 [63]	Yes, mothers of infants who weighed less than 1500 g at birth, Ontario, Canada	Yes, standardized interviews using the Standard Gamble technique to elicit preferences for 5 pediatric hypothetical health states with varying disabilities<br>Yes, quantitative	Closed questions<br>Externally framed analysis
Schmidt 1999 [57]	Yes, premature infants were eligible for enrollment in a placebo-controlled trial of antithrombin therapy, a university medical centre, Canada	Yes, physical health status of newborns with parents who consented or refused<br>Yes, quantitative analysis	No questions<br>Externally framed analysis
Sharma 2011 [64]	Yes, women with at least one prior Caesarean in the last 18 months who were eligible to undergo a trial of labour for a subsequent pregnancy, Oregon, USA.	Yes, 45 minutes using a computerized evidence-based decision aid based<br>Yes, quantitative comparison of two decision aid models	Closed questions<br>Externally framed analysis
Simon 2006 [60]	Yes, parents of children eligible for RCTs of leukemia treatments, Maryland, USA.	Yes, observed & recorded informed consent sessions, and interviews<br>Yes, quantitative analysis of references to altruism related to RCT participation	Open and closed questions<br>Externally framed analysis
Singhal 2002 [26]	Yes, parents of newborn babies in NICU, in a large tertiary referral center and teaching hospital, Canada	Yes, questionnaire with graded responses and five research scenarios	Closed questions<br>Externally framed analysis
Singhal 2004 [41]	Yes, doctors and nurses in a large tertiary care center in western Canada	Yes, questionnaire with 20 scaled items and five case scenarios	Closed questions<br>Externally framed analysis
Snowdon 1997 [48]	Yes, parents of critically ill newborns who	Yes, in-depth interviews at 47-140 weeks after birth	Open questions<br>Emergent themes

	consented to enrolling in ECMO trial, UK.	Yes, thematic analysis with Atlas-ti	
Snowdon 1998 [33]	Yes, parents whose baby had participated in ECMO trial, and they had received trial results, UK	Yes, in-depth interviews<br>Yes, thematic analysis with Atlas-ti	Open questions<br>Emergent themes
Snowdon 2004 [42]	Yes, neonatologists and neonatal pathologists associated with five UK trials	Yes, semi-structured interviews<br>Yes, thematic analysis with Atlas-ti	Open questions<br>Emergent themes
Snowdon 2004 [39]	Yes, bereaved parents from five UK neonatal units	Yes, in-depth interviews<br>Yes, thematic analysis with Atlas-ti	Open questions<br>Emergent themes
Snowdon 2006 [28]	Yes, parents of babies, most associated with the neonatal trials; some parents refused consent, some babies died, UK	Yes, in-depth interviews<br>Yes, thematic analysis with Atlas-ti	Open questions<br>Emergent themes
Stenson 2004 [35]	Yes, parents of sick newborns enrolled in a randomised trial in the early neonatal period, Scotland	Yes, a short questionnaire using Likert scales<br>Yes, quantitative analysis	Closed and open questions<br>Externally framed analysis
Ward 2009 [27]	Yes, in NICU, in USA, mid-Atlantic hospitals	Yes, in-depth interviews;<br>Yes, qualitative content analysis	Open questions, themes developed from the data
Zupancic 1997 [21]	Yes, parents who had recently given or declined consent to one of three controlled trials in the NICU, Canada	Yes, questionnaire using Likert scales<br>Yes, quantitative analysis	Closed questions<br>Externally framed analysis
